# The effect of neuronal conditional knock-out of peroxisome proliferator-activated receptors in the MPTP mouse model of Parkinson’s disease

**DOI:** 10.1016/j.neuroscience.2015.05.048

**Published:** 2015-08-06

**Authors:** R.B. Mounsey, H.L. Martin, M.C. Nelson, R.M. Evans, P. Teismann

**Affiliations:** a School of Medical Sciences, University of Aberdeen, Aberdeen, United Kingdom; b Institute of Molecular Medicine, University of Leeds, Leeds, United Kingdom; c Gene Expression Laboratory, Salk Institute, La Jolla, CA, USA

**Keywords:** 6-OHDA, 6-hydroxydopamine, ANOVA, analysis of variance, DOPAC, 3,4-dihydrophenylacetic acid, HPLC, high-performance liquid chromatography, IL, interleukin, iNOS, inducible nitric oxide synthase, LPS, lipopolysaccharide, MPP, 1-methyl-4-phenylpyridinium, MPTP, 1-methyl-4-phenyl-1,2,3,6-tetrahydropyridine, PBS, phosphate-buffered saline, PBS-T, phosphate-buffered saline-Triton X, PD, Parkinson’s disease, PPAR, peroxisome proliferator-activated receptor, RT-PCR, reverse transcription polymerase chain reaction, SNpc, substania nigra pars compacta, TH, tyrosine hydroxylase, TNF-α, tumor necrosis factor-alpha, Parkinson’s disease, MPTP, neurodegeneration, peroxisome proliferator-activated receptor

## Abstract

Activation of peroxisome proliferator-activated receptors (PPARs), namely PPARγ and PPARδ, has been shown to provide neuroprotection in a number of neurodegenerative disorders, such as Alzheimer’s and Parkinson’s disease (PD). The observed neuroprotective effects in experimental models of PD have been linked to anti-oxidant and anti-inflammatory actions. This study aimed to analyze the full influence of these receptors in neuroprotection by generating a nerve cell-specific conditional knock-out of these receptors and subjecting these genetically modified mice to the 1-methyl-4-phenyl-1,2,3,6-tetrahydropyridine (MPTP) neurotoxin to model dopaminergic degeneration. Mice null for both receptors show the lowest levels of tyrosine hydroxylase (TH)-positive cell bodies following MPTP administration. Presence of one or both these receptors show a trend toward protection against this degeneration, as higher dopaminergic cell immunoreactivity and striatal monoamine levels are evident. These data supplement recent studies that have elected to use agonists of the receptors to regulate immune responses. The results place further importance on the activation of PPARs and the neuroprotective roles these have in inflammatory processes linked to neurodegenerative processes.

## Highlights

• We investigate the role of PPARγ and PPARδ in a model of Parkinson’s disease.
• Conditional neuronal knockout of PPARγ or PPARδ did not increase susceptibility to MPTP.
• Conditional double knockout of neuronal PPARγ and PPARδ did not enhance MPTP toxicity significantly.
• Glial PPAR expression seems more important to confer neuroprotection.

## Introduction

Peroxisome proliferator-activated receptors (PPARs) are a family of nuclear ligand-activated transcription factors controlling a variety of genes with roles in lipid metabolism, insulin sensitivity, fatty acid transport and regulation of inflammation. They do this by binding to specific peroxisome proliferator response elements in enhancer sites of target genes. Initially identified in *Xenopus laevis*, there are three mammalian isoforms – PPARα, PPARδ and PPARγ – each with different tissue expression patterns and ligand affinities (Desvergne and Wahli, 1999).

Parkinson’s disease (PD) is a chronic progressive disorder, characterized by the loss of dopaminergic neurons in the nigrostriatal pathway (Dauer and Przedborski, 2004), with symptoms including bradykinesia, resting tremor and postural instability. This pattern of cell death can be reliably replicated using the 1-methyl-4-phenyl-1,2,3,6-tetrahydropyridine (MPTP) neurotoxin. In the majority of cases the cause of the disease is unknown, while the full pathology of the disease is not understood. However, certain processes have been implicated in the death of neurons, including inflammation, as shown through increased glial activity and astrogliosis in PD brains (McGeer et al., 1988), while MPTP also causes pathogenic upregulation of the immune response (Kurkowska-Jastrzebska et al., 1999). PPARγ agonists provided neuroprotection in the MPTP (Breidert et al., 2002; Dehmer et al., 2004; Lecca et al., 2015; Pisanu et al., 2014; Barbiero et al., 2014) and 6-hydroxydopamine (6-OHDA) model of PD (Laloux et al., 2012; Sadeghian et al., 2012). The PPARγ agonist pioglitazone has been shown to inhibit MPTP- and lipopolysaccharide (LPS)-induced neuronal nuclear factor kappaB activation (Dehmer et al., 2004; Lecca et al., 2015), and PPARγ agonists reduced MPTP-induced tumor necrosis factor-alpha (TNF-α and interleukin (IL)-1β expression (Pisanu et al., 2014), and LPS-induced neuronal cyclo-oxygenase-2, TNF-α, IL-1β and IL-6 expression, thus providing protection (Luna-Medina et al., 2005).

However, the picture is not quite as clear for PPARδ, as an agonist of this receptor had no effect in the 6-OHDA model (Sadeghian et al., 2012), but provided neuroprotection in the MPTP-model of PD (Iwashita et al., 2007; Martin et al., 2013). In general, PPARδ agonists seem to be capable of protecting against oxidative stress and neuroinflammation (reviewed in (Schnegg and Robbins, 2011)).

Herein, we wanted to address the role of neuronal PPAR expression in the neurodegenerative process of PD using the MPTP-model. Mice with neuron-specific disruption of PPARγ and PPARδ coding regions were administered MPTP and measures of dopaminergic neuron survival assessed, thereby evaluating the role of these receptors in neuroprotection.

## Experimental procedures

### Generation of conditional knock-outs

Mice null for one or both of PPARδ and PPARγ were generated using the Cre-lox technique, under the control of a neuronal promoter (Barak et al., 1999, 2002). PPARδ-null, PPARγ-floxed and Nestin Cre mice were bred onto a wild-type C57Bl/6 background and cross-bred to generate combinations of PPARγ-null with or without Nestin Cre and PPARδ-null with or without Nestin Cre. These generations were then cross-bred to produce a double knock-out: PPARγ-null-PPARδ-null with Nestin Cre (PPARγ^ck−/−^/PPARδ^ck−/−^), before the resultant double-knock-out mice were bred together to increase the proportion of these with each generation. Nestin Cre-negative mice were added at alternate generations to maintain the optimum health of animals. It was ensured that littermates did not breed together.

**Fig. 1:**
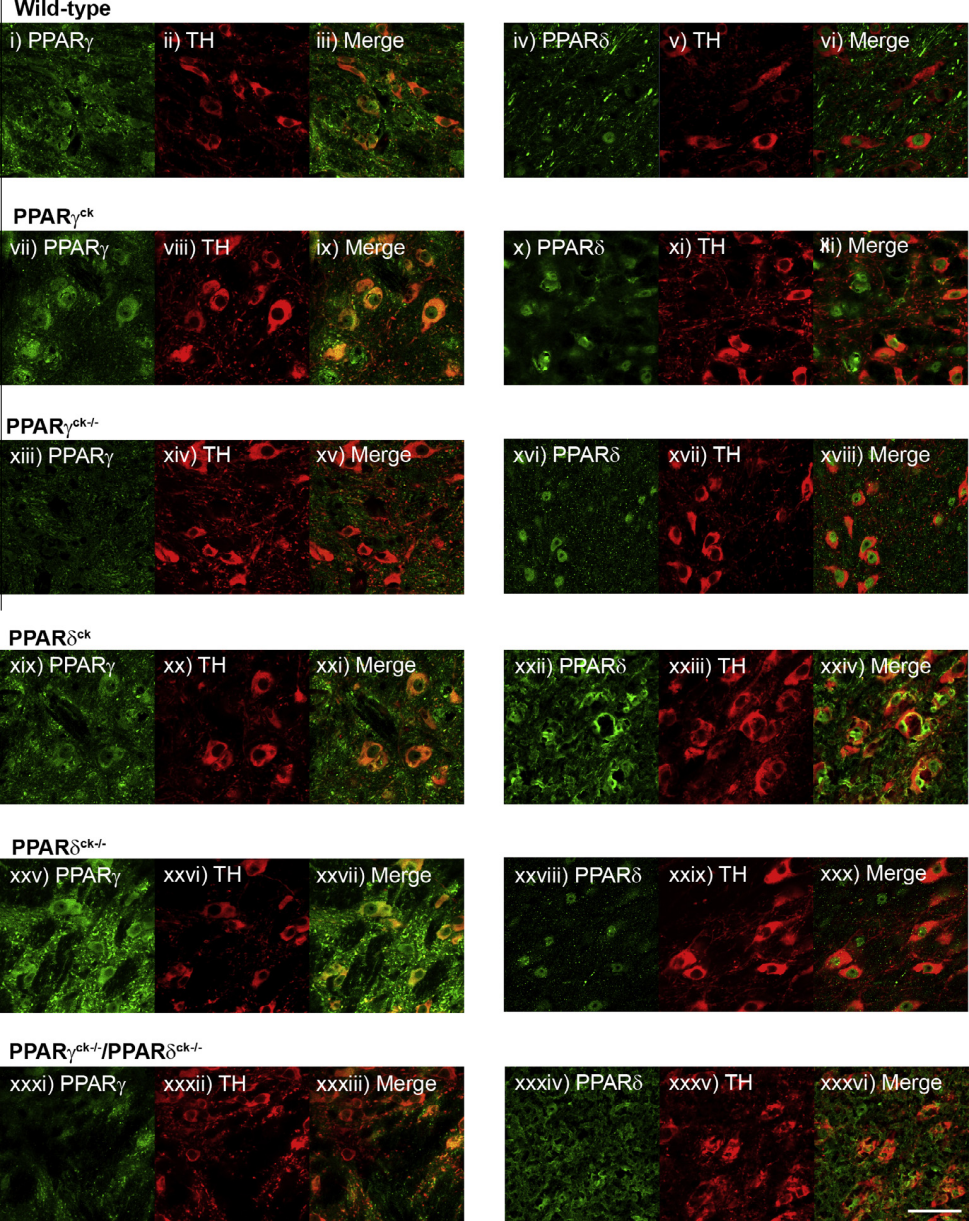
Immunolocalisation of PPARγ and PPARδ in the SNpc of genetically altered mice following MPTP treatment. Double immunofluorescence confirms the presence of PPARγ (i–iii; green) and PPARδ (iv–vi; green) with TH (red) in wild-type mice. The receptors remain visible when the Cre protein is expressed without the gene being excised (PPARγ: vii–ix; PPARδ: xvi–xviii). Fluorescence of PPARγ is greatly reduced in the conditional knock-out (xix–xxi), while PPARδ remains unaffected (xxii–xxiv). PPARδ shows a stronger presence in its knock-out model but expression appears reduced (xxviii–xxx), while PPARγ is unaffected (xxv–xxvii). In the double knock-out images expression of TH in dopaminergic neurons seems reduced and neurons show a change of morphology (xxxi–xxxiii and xxxiv–xxxvi). Scale bar = 50 μm. (For interpretation of the references to color in this figure legend, the reader is referred to the web version of this article.)

**Fig. 2:**
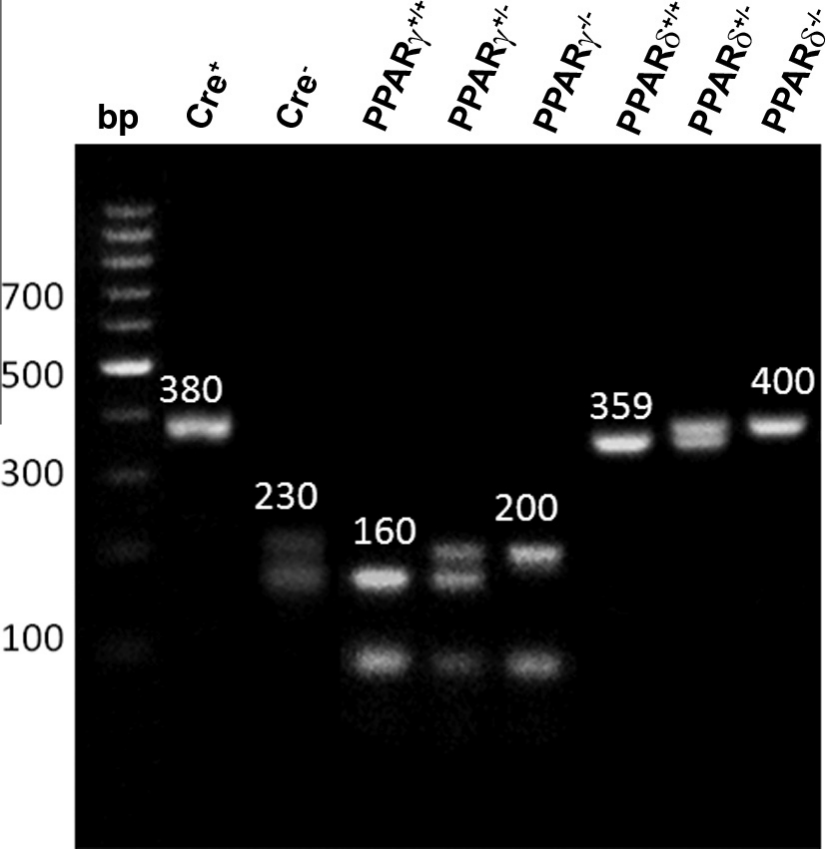
DNA electrophoresis of transgenic mice. Genotypes of mice were ascertained by measuring the band size of DNA extracts following PCR in the presence of specific primers.

Genotypes were confirmed by reverse transcription polymerase chain reaction (RT-PCR) using the Go Taq amplification system (Promega, Southampton, UK), as per manufacturer’s instructions, following DNA extraction from ear clips and immunofluorescence staining of the receptors (Figs. 1 and 2).

Earclips of mice were taken and DNA samples extracted using DNAreleasy (Anachem, Luton, UK), following manufacturer’s instructions. Genotyping was performed by RT-PCR using the Go Taq amplification system (Promega, Southampton, UK) as per manufacturer’s instructions, with reaction mixture details in Table 1.

The information regarding primers and their sequences, annealing temperatures and electrophoresis bands are detailed in Table 2. All PCR cycles were subject to a hot-start (94–95 °C). For Nestin Cre cycle PCR conditions were 35 cycles of 94 °C for 30 s, 51 °C for 60 s and 72 °C for 60 s. For PPARγ and PPARδ conditions were 35 cycles of 94 °C for 20 s, 60 °C for 30 s and 71.5 °C for 70 s. Products were electrophoresed on a 2% agarose gel and visualized with ethidium bromide (final concentration 0.1%) using the Alpha Innotech digital imaging system (San Leandro, CA, USA).

Mutant mice, in terms of their phenotype, were generally smaller at birth. This was a pattern noticed with PPARδ KO mice, so the phenotype difference is likely due to a downstream effect of this receptor.

### Animal treatments

Twelve-week-old male C57Bl/6 mice, PPARγ and PPARδ-genetically altered mice received intraperitoneal injections of MPTP·HCl (30 mg/kg free base), dissolved in 0.9% saline solution, one injection per day for five consecutive days, before being sacrificed by decapitation 21 days after the last injection. Control mice received saline only. This treatment was in accordance with the published guidelines (Jackson-Lewis and Przedborski, 2007). All procedures were in accordance with the Animals (Scientific Procedures) Act 1986 and were approved by the Home Office, Dundee, UK.

### Immunohistochemistry and stereology

Brains were fixed and processed for immunostaining as described previously (Teismann et al., 2003; Sathe et al., 2012). Primary and secondary antibodies used were rabbit anti-tyrosine hydroxylase (TH) (1:1000; Millipore, Watford, UK) and biotinylated goat anti-rabbit (1:200; Vector Laboratories, Peterborough, UK). Immunostaining was visualized with 3,3′-diaminobenzidine (Sigma–Aldrich, Poole, UK; 25 mg in 50 ml 0.1 M Tris GN pH 7.6 with 100 μl ammonium chloride (40 mg/200 μl Tris GN), 150 μl glucose oxidase (30 mg/10 ml Tris GN) and 400 μl glucose (200 mg/800 μl Tris GN)). Sections were counterstained with Nissl reagent (thionin). The total number of TH-positive neurons and Nissl-positive cells in the substantia nigra pars compacta (SNpc) was counted in the various groups of animals at 21 days after the last MPTP or saline injection using the unbiased optical fractionator method, as described previously (Teismann et al., 2003; Sathe et al., 2012). Counting of TH-positive cells was performed using regular light microscopy (AxioImager M1, Carl Zeiss, Cambridge, UK) and the optical fractionator method (West, 1993) (Stereo Investigator Version 7, BMF Bioscience, Magdeburg, Germany), while the observer was blinded to the subjects’ identity.

Striatal density of TH immunoreactivity was determined as described previously (Wu et al., 2002) and assessed on scans (Hewlett Packard Scanjet G3110, Bracknell, Berkshire, UK) of the sections using Scion Image (Version 4.0.3.2, Scion Corporation, MD, USA).

### Immunofluorescence

Immunofluorescent staining was performed as described (Teismann et al., 2003; Sathe et al., 2012). Sections were washed three times for 5 min with 0.1% Triton X in 0.1 M phosphate-buffered saline (PBS), before non-specific binding was blocked with 10% normal goat serum in 0.1 M PBS-Triton X (PBS-T). Sections were incubated overnight at 4 °C in 0.1 M PBS-T with primary antibodies as follows: rabbit anti-TH (1:1000; Millipore), PPARγ (1:100, Alexis Biochemicals, San Diego, CA, USA), PPARδ (1:250, Abcam, Cambridge, UK). Following further washes, immunostaining was visualized with Alexa Fluor 488 anti-rabbit or anti-mouse (1:300; Molecular Probes, Eugene, OR, USA) or cy-3 anti-rabbit or anti-mouse (1:200; Jackson ImmunoResearch, West Grove, PA, USA) antibodies. After three final washes, sections were mounted on slides with Mowiol-DABCO. Immunostaining was visualized by confocal microscopy (LSM 510 or LSM 700, Carl Zeiss).

### High performance liquid chromatography (HPLC)

HPLC with electrochemical detection was used to measure striatal levels of dopamine and 3,4-dihydroxyphenylacetic acid (DOPAC) using a method that has been described (Nuber et al., 2008).

### Statistical analysis

Data were analyzed in IBM SPSS Statistics Version 21 for Windows (Hampshire, UK). All values are expressed as the mean ± SEM. In the case of TH-numbers, Nissl counts and striatal optical density normal distribution of the data was tested and confirmed with the Shapiro–Wilk test. Homogeneity of variance was assessed by Levene’s Test for Equality of Variance. For data sets, two-way analysis of variance (ANOVA) was used to assess differences among means, with genotype and treatment as the independent factor. When ANOVA showed significant differences, Tukey’s post hoc testing was used to make comparisons between means. As not all groups are reported, we also assessed the differences among means with genotype as the independent factor in the two different treatment groups, followed by Newman–Keuls post hoc test. In the case of dopamine and DOPAC levels normal distribution of the data was tested and confirmed for logarithmized values with the Shapiro–Wilk test. Outliers were eliminated based on descriptive statistics performed by SPSS. Data are represented in non-log format for better understanding. When ANOVA showed significant differences, Tukey’s post hoc testing was used to make comparisons between means. As not all groups are reported, we also assessed the differences among means with genotype as the independent factor in the two different treatment groups, followed by Newman–Keuls post hoc test. The null hypothesis was rejected at the 0.05 level.

## Results

**Fig. 3:**
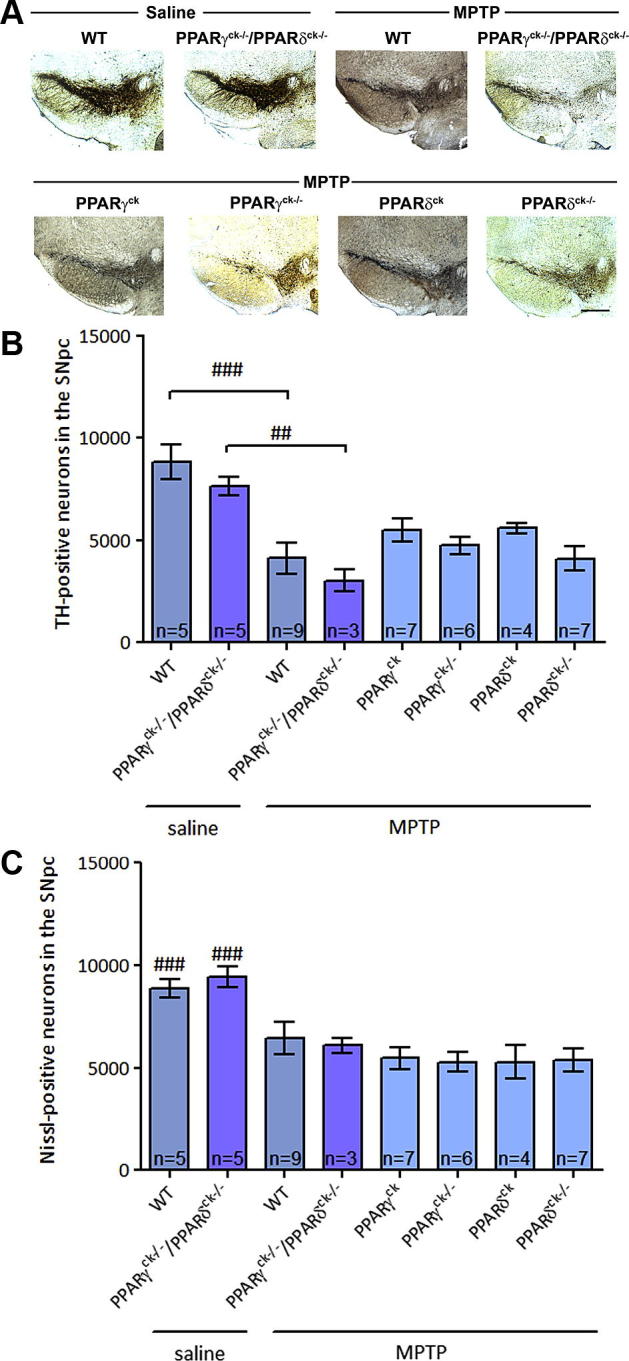
Effect of PPARγ and/or PPARδ conditional knock-out on MPTP toxicity. (A) Representative photomicrograph images of saline- and MPTP-treated SNpc sections. Scale bar = 200 μm. (B) MPTP significantly reduces levels of TH-positive neurons in the SNpc of both wild-type (WT) and double knock-out (PPARγ^ck−/−^/PPARδ^ck−/−^) mice. When this group is compared to PPARγ or PPARδ single conditional knock-out mice there is no significant change. There is also no difference between PPARγ^ck−/−^/PPARδ^ck−/−^ and mice with the target genes floxed (PPARγ^ck^ and PPARδ^ck^). (C) Loss of Nissl-positive cells confirmed that the loss of TH-positive neurons corresponds to an actual loss of neurons. Data are mean ± SEM, *n* = 3–9 per group. ^**^p < 0.01, ^***^p < 0.001, compared to saline-treated group of same genotype; ^##^*p *< 0.01, ^###^*p *< 0.001, compared to MPTP-treated groups (one-way ANOVA followed by Newman–Keuls post hoc test) (TH – tyrosine hydroxylase; SNpc – substantia nigra pars compacta).

To assess the precise contribution that the two receptors may have in a neuroprotective mechanism in PD, the transgenic mice were injected with the MPTP neurotoxin. Numbers of TH immunoreactive and Nissl-positive cells were stereologically counted (Fig. 3). A two-way ANOVA was conducted to assess the effect of genotype and treatment on numbers of TH-immunoreactive neurons and Nissl-positive cells. There was a statistically significant interaction between genotype or treatment on numbers of TH-positive neurons as well as Nissl-positive cells. To assess the effect of genotype on MPTP-induced cell loss, a one-way ANOVA was performed and, as expected, MPTP caused a significant degeneration of TH-positive neurons in both wild-type (*p* < 0.001, a one-way ANOVA followed by Newman–Keuls post hoc test) and PPARγ^ck−/−^/PPARδ^ck−/−^ mice (*p* < 0.01, a one-way ANOVA followed by Newman–Keuls post hoc test), compared to their saline-treated controls of the same genotype. MPTP also caused a significant degeneration of Nissl-positive cells in both wild-type (*p* < 0.001, a one-way ANOVA followed by Newman–Keuls post hoc test) and PPARγ^ck−/−^/PPARδ^ck−/−^ mice (*p* < 0.001, a one-way ANOVA followed by Newman–Keuls post hoc test), compared to their saline-treated controls of the same genotype. No significant change in MPTP-induced cell death was evident 21 days after toxin treatment when double knock-out mice were compared with mice with a single knock-out of PPARγ or PPARδ or PPAR floxed mice without a target gene excised. It is possible to argue that higher numbers of PPARγ^ck−/−^/PPARδ^ck−/−^ MPTP-treated brains could yield statistically significant results in this comparison since a pattern seems to show that double knock-out mice are slightly more sensitive to toxin-induced cell death.

**Fig. 4:**
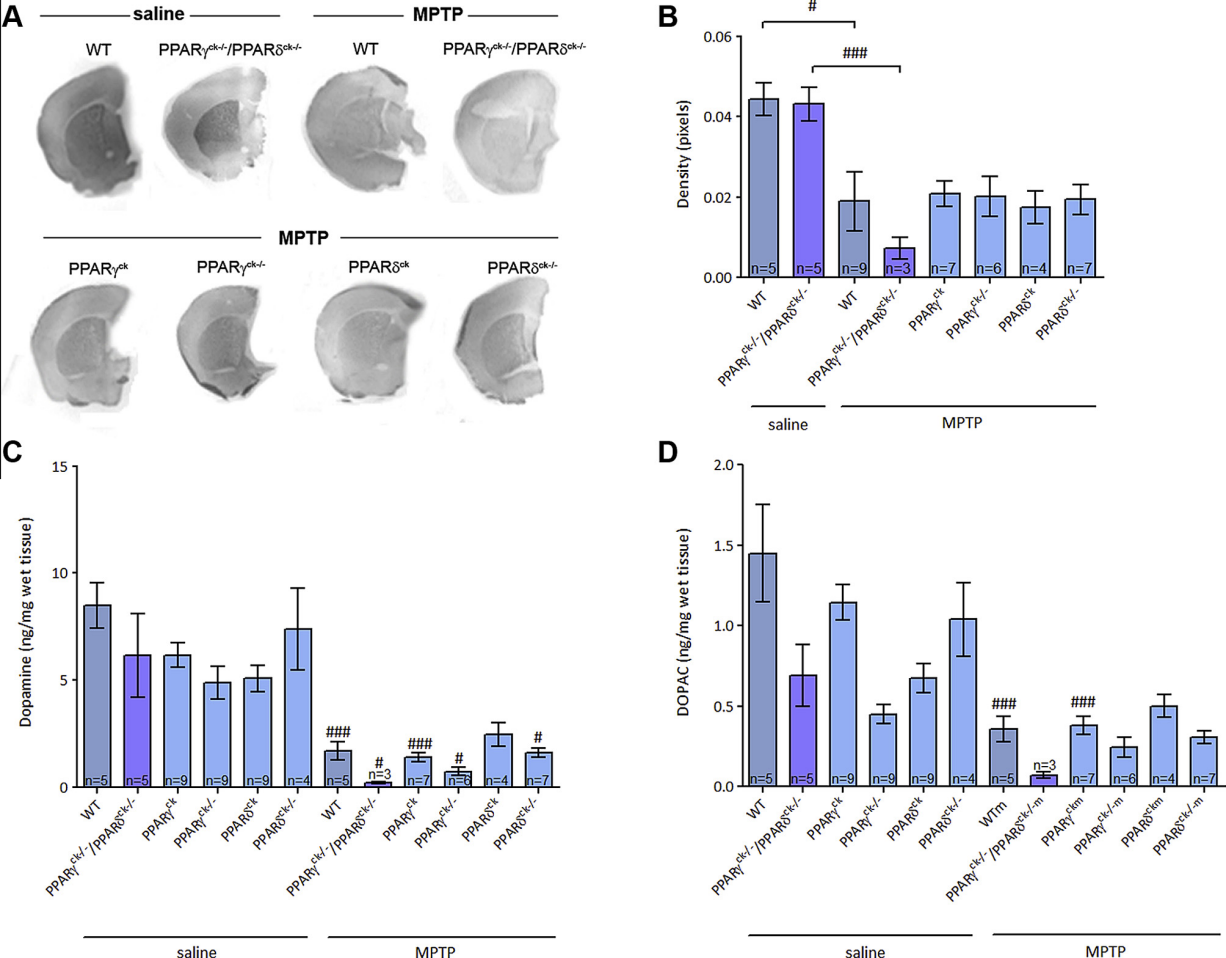
Striatal dopaminergic innervation and dopamine and DOPAC content of PPARγ and PPARδ genetically manipulated mice. (A) Representative scanned images of striatal sections following saline or MPTP administration. (B) MPTP significantly reduces the density of striatal sections when the double knock-out and wild-type mice are compared with the corresponding saline-treated sections. Single knock-out or mice with the target gene floxed without PPARγ or PPARδ excised show no significant variation from the PPARγ^ck−/−^/PPARδ^ck−/−^ mice. (C) Genotype does not significantly affect striatal dopamine levels but wild-type, PPAR^ck^ and PPAR^ck−/−^ values are reduced following MPTP administration. (D) Striatal DOPAC levels are also unaffected by genetic manipulation but wild-type and PPAR^ck^ values are again reduced following MPTP administration. Data are mean ± SEM, *n* = 3–9 per group. ^#^*p *< 0.05, ^###^*p < *0.001, compared to saline-treated group of same genotype (a one-way ANOVA followed by Newman–Keuls post hoc test).

To measure the impact on striatal fibers which innervate these bodies, TH density staining was assessed. MPTP reduced TH innervation of the striatum across all genotypes. The MPTP-treated double knock-out brains have the lowest mean striatal density. Following the pattern of nigral TH immunoreactivity (Fig. 4A, B), the striatal densities of floxed and single PPARγ or PPARδ knock-out mice appear higher, but the differences are not significant.

Levels of striatal monoamine were determined using HPLC analysis (Fig. 4C, D). Across values of dopamine and its metabolite DOPAC, significant differences were not found between MPTP-treated PPARγ^ck−/−^/PPARδ^ck−/−^ mice and those with one or both of PPARγ and PPARδ genes left intact, but the mean value for the double knock-out mice remains lowest of all treatment groups. There was a statistically significant interaction between genotype or treatment on striatal dopamine content (a two-way ANOVA followed by Tukey post hoc test). To assess the effect of genotype alone on MPTP-treatment a one-way-ANOVA was performed. There are significant differences in dopamine levels between saline-treated wild-type (*p* < 0.001, a one-way ANOVA followed by Newman–Keuls post hoc test), PPARγ^ck^ and PPARγ^ck−/−^ (*p* < 0.01, a one-way ANOVA followed by Newman–Keuls post hoc test) and their MPTP-infused littermates, while DOPAC levels are diminished when MPTP is administered to wild-type and PPARγ^ck^ mice compared to their saline-treated equivalents (*p* < 0.01, a one-way ANOVA followed by Newman–Keuls post hoc test).

Staining was performed on mice treated with MPTP of all genotypes to give an indication of knock-out success (Fig. 1). Both receptors are clearly visible following double immunofluorescence of PPARγ (i–iii; green) or PPARδ (iv–vi; green) with TH (red) in wild-type mice. PPARγ is present in a peri-nuclear location and PPARδ is found in the nuclei of neurons. This presence is maintained when the Cre recombinase protein is expressed without the gene being excised (PPARγ: vii–ix; PPARδ: xxii–xxiv). When mice null for the gene are compared with Cre^+^ mice, the expression of the relevant gene is diminished. Fluorescence of PPARγ is greatly reduced in conditional knock-out mice (xiii–xv), while PPARδ remains unaffected (xvi–xviii). PPARδ appears to remain at a relatively high expression level in its knock-out model (xxviii–xxx), but at a diminished level, while PPARγ is unaffected (xxv-xxvii). This may be due to a form of Cre mosaicism in this particular group, difficult to detect with standard genotyping. In the double knock-out images, both receptors, particularly PPARγ, show greatly reduced expression and TH cell morphology appears to have changed (xxxi–xxxiii and xxxiv–xxxvi). Overall TH-positive neuron numbers appear lower, as would be predicted from the stereological data shown above.

## Discussion

This study aimed to assess the contributions of PPARγ and PPARδ in MPTP toxicity. Previous work through the use of receptor-specific ligands has delineated the neuroprotective effects that activation of these receptors has in several models of neurodegeneration. There is evidence that they are likely to work through negative modulation of immune responses through the inhibition of pro-inflammatory cytokine release (Bishop-Bailey and Bystrom, 2009). To consider the relative roles of the two receptor subtypes and their contributions to such a mechanism we generated single and double knock-out mice of both PPAR isoforms. It was necessary to generate double knockouts of PPARγ and PPARδ as PPAR isoforms are known to be subject to functional compensation (Patsouris et al., 2006). Importantly, this compensatory change is known to occur in neurons (Gonzalez-Aparicio et al., 2011). Conditional knock-outs were required as a complete knock-out of either receptor is lethal. Studies by Barak and colleagues have investigated the viability of PPARγ^−/−^ and PPARδ^−/−^ mice (Barak et al., 1999, 2002). Inducing PPARγ deficiency through homologous recombination causes death at two independent developmental points, both of which result in embryonic death by day 10 (Barak et al., 1999). Similarly, PPARδ-deficiency results in a high degree of embryonic lethality (over 90%) with surviving mice smaller then wild-type counterparts, while offspring of these mice typically do not survive to full term due to placental defects (Barak et al., 2002). It was subsequently found that PPARδ is a critical molecular signaling link during the processes of maternal implantation and decidualization, with embryonic expression of the receptor required for placentation (Wang et al., 2007). The Cre-*lox P* method, a technique pioneered by Sauer and Henderson (1988), was utilized to produce tissue-specific knock-outs of the gene products to avoid the lethality which affects complete PPARγ and PPARδ knock-outs. Still, by using the knockout technique, mutation occurs systemically. Specifically silencing PPARδ and/or PPARγ in the SNpc or striatum could be a further useful approach to delineate the function of these PPAR isoforms.

Offspring of several genotypes were administered MPTP before assessing neurodegeneration. Numbers of TH-positive cells were lowest in the PPARγ^ck−/−^/PPARδ^ck−/−^ group of mice, although this number did not vary significantly from that shown by single knock-out or floxed mice. The relatively even number of cells across the PPARγ^ck−/−^ and PPARδ^ck−/−^ groups may indicate equally significant contributions to processes underlying overall neuron survival from activation of these receptor subtypes. Furthermore, a lower mean TH-positive cell count among PPARγ^ck−/−^/PPARδ^ck−/−^ mice potentially indicates a degree of functional compensation that may acquire increased importance when expression of one receptor is lower than physiological levels. As PPARγ and PPARδ can also be expressed by glial cells, it seems more likely that the expression of PPARs on these cells is more relevant to the overall effect of PPAR-mediated effects in the MPTP-model. The trend observed in neuron cell bodies was adhered to in other measurements of dopaminergic cell loss. The density of TH-positive fiber projection to the striatum showed an identical pattern to that of nigral TH-positive neurons, while striatal dopamine and DOPAC levels, measured by HPLC, demonstrated similar results. All genotypes express lower dopamine levels, although not to a significant extent. This could be an effect of the genes involved which, despite not being active, might still have an impact on overall dopamine content. Wild-type mice show similar levels of TH-positive cells compared to the single receptor knock-out or floxed mice. This may be due to a functional compensation of the PPAR isoforms to levels where physiological neuroprotective mechanisms are maintained. Further studies should address whether the observed changes also translate to functional changes using appropriate behavioral tests.

It has been shown that heterozygous PPARδ mice maintain levels of protein relative to that of wild-type mice despite having approximately half the PPARδ mRNA, thereby indicating PPARδ has a vital function in the basal activity of neurons (Martin et al., 2013). The importance of PPARδ has been proposed previously, with evidence the isoform acts as a ‘gateway receptor’, as stable expression of the PPARδ inhibits that of PPARγ and modulates its function (Shi et al., 2002). As noted above, the levels of dopaminergic cell survival were relatively equal in PPARγ^ck−/−^ and PPARδ^ck−/−^ mice. This indicates that PPARδ may play no particular importance in the regulation of inflammation over the PPARγ isoform.

However, there is an absence of statistical significance in the results, likely due in part to the low numbers of PPARγ^ck−/−^/PPARδ^ck−/−^, a result of the difficulty in producing these mice in the time-frame of the study. The work, nonetheless, provides compelling initial genetic evidence that backs up pharmacological studies supporting the importance in PPAR activation in neuronal survival. Pharmacological antagonism of both receptors has independently proven to reduce cell survival. The selective PPARδ antagonist GSK0660 can exacerbate 1-methyl-4-phenylpyridinium (MPP)^+^-induced cell death *in vitro* (Martin et al., 2013). A selective antagonist of PPARγ, bisphenol A diglycidyl ether, causes deteriorating clinical performance in a model of multiple sclerosis (Raikwar et al., 2005). Another antagonist at this receptor subtype, GW9662, augmented MPTP-induced loss of TH-positive neuron in mice (Martin et al., 2012), demonstrating that activation of these receptors may be important in protection against inflammatory insult. Indeed, there are many studies providing signs that PPARγ and PPARδ activation is important in mediating neuroprotection. Investigations of the PPARγ agonists pioglitazone (Breidert et al., 2002; Dehmer et al., 2004), rosiglitazone (Schintu et al., 2009; Martin et al., 2012; Pisanu et al., 2014) and the non-thiazolidinedione MDG548 (Lecca et al., 2015) have provided evidence that these agents can attenuate MPTP-induced neuronal loss in the SNpc. The thiazolidinedione pioglitazone is able to restore mitochondrial function following administration of the bacterial endotoxin LPS in rats (Hunter et al., 2007). This particular action may be through an upregulation of anti-apoptotic protein Bcl-2 (Fuenzalida et al., 2007), which can inhibit the opening of mitochondrial permeability pores (Zorov et al., 2009). Furthermore, additional experiments need to demonstrate if PPAR agonists provide protection in a regional-specific model of PD, such as the lentiviral-based delivery of α-synuclein (Lo et al., 2002), thus demonstrating the relevance of PPARs as a potential neuroprotective therapy. It would also be useful to assess the impact of silencing these receptors in a specific region, such as SNpc or even the striatum.

In all cases protection occurs alongside a reduction in the immune response as microglial and astrocyte activation is reduced. PPARγ agonists can reduce inflammatory responses including production of TNFα and inducible nitric oxide synthase (iNOS) (Breidert et al., 2002). Both these processes have been implicated in the death of dopaminergic neurons (Boka et al., 1994; Hunot et al., 1996). In addition, PPARγ activation led to a reduction of MPTP-induced nitrotyrosine levels, a marker for NO-mediated damage (Dehmer et al., 2004), and reduced MPTP-mediated increase in iNOS expression (Lecca et al., 2015). A possible antioxidant role of PPARγ activation is supported by our own group, as we demonstrated that rosiglitazone attenuates reactive oxygen species (ROS) formation induced by MPP^+^ *in vitro* (Martin et al., 2012). This may be through upregulation of superoxide dismutase (SOD) and catalase expression (Jung et al., 2007). Alterations in PPAR expression also supplement the hypothesis that the presence of the PPARγ and PPARδ subtypes together is important in neuroprotection. Both the mRNA and protein levels of PPARγ in the ventral midbrain are upregulated 7 days after MPTP treatment (Martin et al., 2012). Similarly, PPARδ mRNA and protein levels show an immediate upregulation in the striatum (Martin et al., 2013). These expression alterations may represent an endogenous defence mechanism against the inflammatory and oxidative insults of MPTP – a mechanism that PPARγ^ck−/−^/PPARδ^ck−/−^ mice are likely devoid of, leading to reduced neuron survival. Furthermore, endogenous ligands of the PPARs may have a role. The structure of these receptors allow for the binding of an array of ligands, including fatty acids, eicosanoids and steroids. The impact these could have on this mechanism is not currently known.

It would be interesting to investigate the protective abilities of PPAR agonists in knock-out animals to test whether the benefits shown by these agents are dependent upon receptor activation or can be initiated independently of the receptor.

Biological effects following administration of agonists that are not dependent upon PPARγ activation, such as antioxidant benefits, have a significant impact upon neuroprotection (Davies et al., 2001; Chintharlapalli et al., 2005; Wang et al., 2011; Martin et al., 2012). The use of agonists may have an influence on cellular metabolic function as pioglitazone could increase glucose uptake by cells, thereby increasing their resistance to MPTP (Breidert et al., 2002). It remains to be seen whether these same pro-inflammatory mediators are inhibited without the direct action of the ligand, but this study further underlines the importance of PPAR pathways in models of neuronal degeneration.

## Conclusion

Neuronal PPARγ or PPARδ does not seem to counteract MPTP-induced toxicity. Different aspects need to be taken into account to explain the findings. The results might be due to the fact that ablation of the neuronal receptors was not 100% complete, but were below the level to be picked up using PCR and immunohistochemistry. Since PPARs are also expressed on glial cells, it may be argued that the main protective role of PPARs is played by glial receptors rather than neuronal, an aspect which needs further investigation.

## Figures and Tables

**Table 1:** RT-PCR reaction mixture for genotyping

Reaction mixture
4 μl 5× Go Taq Green reaction buffer
2 μl 2 mM dNTPs
1 μl of each primer
0.1 μl of Taq polymerase (5 U/μl)
1 μl DNA
Total volume adjusted to 20 μl with sterile distilled water

**Table 2:** Primers for genotyping. (Primers were purchased from Sigma–Aldrich)

Target	Primer	Type	Sequence (5′-3′)	Annealing temp. (°C)	Electrophoresis band (bp)	Reaction product
Nestin Cre	Wild-type	Forward primer	CTAGGCCACAGAATTGAAAGA		230	wt
		Reverse primer	GTAGGTGGAAATTCTAGCATCATCC	51		
	Transgene	Forward primer	GCGGTCTGGCAGTAAAAACTA		380	Cre^+^
		Reverse primer	GTGAAACAGCATTGCTGTCAC			

PPARγ	lox-PPARg	Forward primer	CTAGTGAAGTATACTATACTCTGTGCAGCC		160	wt
		Reverse primer	GTGTCATAATAAACATGGGAGCATAGAAGC	60	200	PPARγ^−^

PPARδ	Common		GAGCCGCCTCTCGCCATCCTTTCAG		359	wt
	Wild-type specific	–	GGCGTGGGGATTTGCCTGCTTCA		400	PPARδ^−^
	Knock-out specific		GTCGAGAAGTACTAGTGGCCAGTGG			
